# The impact of international care networks on the clinical management of constitutional mismatch repair deficiency (CMMRD): a review of recent developments

**DOI:** 10.1007/s10689-026-00584-x

**Published:** 2026-06-25

**Authors:** Hans F. A. Vasen, Katharina Wimmer, Mariëtte van Kouwen, Léa Guerrini-Rousseau, Daniela Gattini, Lucie Stengs, Uri Tabori, Chrystelle Colas, Anirban Das

**Affiliations:** 1 Department of Gastroenterology and Hepatology, Leiden University Medical Centre, Leiden, The Netherlands; 2 Institute of Human Genetics, Medical University Innsbruck, Innsbruck, Austria; 3 Department of Gastroenterology and Hepatology, Radboud University Medical Center Nijmegen, Nijmegen, The Netherlands; 4 Department of Children and Adolescents Oncology, Gustave Roussy Cancer Center, Université Paris-Saclay, Villejuif, France; 5 Division of Gastroenterology, Hepatology and Nutrition, SickKids, Toronto, Canada; 6 Division of Hematology Oncology, SickKids, Toronto, Canada; 7 The Arthur and Sonia Labatt Brain Tumour Research Centre, The Hospital for Sick Children, Toronto, ON Canada; 8 Department of Genetics, Institute Curie, Paris, France; 9 INSERM U 1339, DRUM team, Paris, France

**Keywords:** Constitutional mismatch repair deficiency, Cancer predisposition syndrome, Diagnostic guidelines, Surveillance protocols, Microsatellite instability, Immune checkpoint inhibitors

## Abstract

Constitutional mismatch repair deficiency (CMMRD) is a rare and likely the most penetrant cancer predisposition syndrome caused by biallelic germline variants in a mismatch repair gene. Patients typically develop a spectrum of malignancies, including brain tumors, gastrointestinal cancers, and hematological neoplasms within the first two decades of life. Since its initial description in 1999, two international consortia, the International Replication Repair Deficiency Consortium (IRRDC) and Care for CMMRD (C4CMMRD), have been established to better understand the syndrome, leading to the creation of diagnostic guidelines and surveillance protocols. This review summarizes recent data on the CMMRD tumor spectrum and discusses the consortia’s updated diagnostic criteria and management guidelines, including novel blood-based assays for detecting constitutional microsatellite instability. Furthermore, we present the initial results and subsequent adjustments to international surveillance protocols. Finally, we discuss the demonstrated efficacy of immune checkpoint inhibitor (ICI) treatment, a key therapeutic advancement for CMMRD patients.

## Introduction

 Lynch syndrome is an autosomal dominant hereditary disorder caused by a heterozygous pathogenic germline variant in one of the DNA mismatch repair (MMR) genes. Carriers of such a variant have an increased risk of developing colorectal carcinoma (CRC), endometrial carcinoma (EC), and various other tumors [1]. More than 25 years ago, patients with pathogenic biallelic MMR germline variants were first described, leading to a syndrome with an autosomal recessive inheritance pattern known as constitutional mismatch repair deficiency syndrome (CMMRD) [2, 3]. The tumor spectrum in CMMRD differs significantly from that in Lynch syndrome, particularly due to the occurrence of brain tumors and hematological malignancies in childhood. Lynch syndrome-associated tumors—such as colorectal carcinomas, small bowel carcinomas, and other malignant conditions— will develop in all patients that reach teenage years/young adulthood.

This review aims to provide a narrative overview of the historical developments in CMMRD, with a particular focus on the activities of two international consortia over the years, and to highlight recent developments in diagnosis and treatment.

## Historical developments

In 1999, a French research group described a remarkable case involving two children from a North African family with Lynch syndrome [2]. Both parents were carriers of the familial pathogenic *MLH1* variant. Two daughters inherited this variant from both their father and mother. The first daughter died at the age of two from non-Hodgkin lymphoma (NHL), while the second developed acute myeloid leukemia at the age of six and a medulloblastoma one year later. Both children had multiple café-au-lait spots. Back-to-back to this publication, a large Turkish family with Lynch syndrome caused by a pathogenic *MLH1* variant was described [3]. In a consanguineous marriage, two daughters were diagnosed with leukemia at the ages of one and two years, respectively, and a third daughter developed NHL at the age of three. Two of the three children exhibited symptoms also seen in neurofibromatosis type 1. Other previously published cases of CMMRD were likely misclassified as Turcot syndrome—defined as the combination of a primary brain tumor and colorectal adenomas or polyposis—before the genetic basis was fully understood [4].

CMMRD is an extremely rare condition. Since the initial reports, over 300 patients with CMMRD have been published [5]. The birth prevalence is estimated to be 1 in a million [6]. Due to the autosomal recessive inheritance pattern, the condition occurs more frequently in families with consanguinity. Therefore, prevalence is higher in countries with high rates of consanguinity or endogamy [7–9], and among indigenous and immigrant communities in other countries without high consanguinity rates [10].

## International consortia

In 2007, Canadian researchers established the International Replication Repair Deficiency Consortium (IRRDC) to advance the understanding of this very rare condition, in particular to clarify the patient phenotype, spectrum of CMMRD cancers, to determine genotype–phenotype associations, surveillance guidelines and treatment recommendations [11]. A total of 160 families were enrolled in the IRRDC between 2007 and 2022 [10]. A first milestone followed in 2012, when the team of Durno et al. published the initial results of surveillance in two sisters with CMMRD [12]. In 2021, they published the long-term results of surveillance in a large series of patients [11] and updated the international surveillance guidelines under the aegis of the AACR Cancer Predisposition Working Group [13]. Novel immune-directed treatment strategies for deadly cancers in these patients were first described in 2016 [14] followed by clinical trials and registry studies that have expanded the treatment options for these patients [15–17].

In 2013, the European consortium ‘Care for CMMRD’ (‘C4CMMRD’) was founded with the aims of improving care for patients with CMMRD. The group established a database for patients from mainly European countries including more than 100 patients. One of the first achievements of the group was the development of criteria for the suspect diagnosis of CMMRD and a formal surveillance protocol in 2014 [18, 19]. American guidelines for monitoring and treatment were later published as well [20, 21]. In 2019, an International Working Group with members of the IRRDC, C4CMMRD and Pediatric Working Group of the AACR developed criteria for the diagnosis of CMMRD [22]. In 2024, ERN GENTURIS developed with C4CMMRD consortium comprehensive CMMRD guidelines, including detailed pathways and criteria to definitely confirm or refute CMMRD in a suspected patient [23].

## Clinical presentation

A characteristic aspect of CMMRD is the frequent occurrence of multiple primary tumors, both in the same organ and in different organs. Table 1 compares the spectrum of common CMMRD tumors from three sources: (1) a systematic review of 146 published cases [18]; (2) the initial cohort of 110 patients from the IRRD Consortium [11]; and (3) the Consortium’s subsequent, expanded series of 201 patients [10], which encompasses the earlier 110-patient cohort.

### Brain tumors

Brain tumors, particularly high-grade gliomas, are the most common malignancy in children with CMMRD (Table 1). The median age at diagnosis is around 9 years. Symptoms typically arise from increased intracranial pressure and manifest as headaches, nausea, vomiting, and double vision. These tumors are known for their aggressive growth, frequently resulting in an advanced stage at the time of diagnosis. The 5-year survival rate for patients with CMMRD who develop symptomatic brain tumors is only 22%-33% [11, 24]. Data from the IRRDC has expanded on the spectrum of brain tumors in CMMRD. Three distinct biological subgroups of high-grade glioma with unique driver mutations impacting clinical outcomes have been recently described, stimulating the development of subgroup-specific treatment approaches [25]. Medulloblastoma and ependymoma which harbor unique biology distinct from sporadic or other germline etiologies have been described [26, 27].

### Hematological malignancies

Hematological malignancies also occur primarily in childhood, with T-cell and B-cell lymphoma (non-Hodgkin lymphoma (NHL)) and acute lymphoblastic leukemia (ALL) being the most common forms. The median age at diagnosis for these malignancies was 6 years in the case reports [18] and around 10 years in the IRRDC-series [10, 11]. (Table 1).

**Table 1:** Tumor spectrum CMMRD

Tumor type	225 tumors (146 patients) Wimmer et al. [18]		193 tumors (110 patients) Durno et al. [11]		339 tumors (201 patients) Ercan et al. [10]	
	*N* (%)	Median age, range (yrs)	*N* (%)	Median age, range (yrs)	*N* (%)	Median age, IQR (yrs)
**Brain tumors**	81 (36%)	9; 2–40	85 (44%)	9.9; 2.3–38.5	173 (51%)	9.7; 6.9–12.9
High grade glioma	58		70		140	
-sPNET*	8		–		–	
Medulloblastoma	7		10		18	
Embryonal tumor (unclassified)	-		5		7	
Other	8		–		8	
**Hematological malignancies**	48 (21.3%)	6; 0.4–21	37 (19%)	10.5; 2.2–29.9	61 (18%)	9.7; 5.3–13.4
Non-Hodgkin lymphoma unspecified	7		1		2	
NHL T-cell/ T-cell lymphoblastic lymphoma	20		18		26	
NHL B-cell / B-cell lymphoma	4		7		14	
(Precursor B-cell, T-cell, unclassified) acute lymphoblastic leukemia	9**		7		12**	
Acute myeloide leukemia	5		4		4	
Other	3		-		3	
**Gastrointestinal cancers**	77 (34.2%)		52 (27%)		75 (22%)	20.1; 13.9–24.9
Colorectal cancer	59***	16; 8–48	37	16; 9–50	59	
Small bowel cancer	18	28; 11–42	11	21; 9–33	12	
Gastric cancer	–		3		3	
Liver cancer	–		1		-	
Pancreatic cancer	-				1	
**Other Lynch syndrome associated tumors**	11 (4.8%)		6 (3%)		10 (3%)	
Endometrial cancer	6	28; 23–44	1	30	-	
Ovarian cancer	1	17	-		-	
Bladder/ureter/renal pelvis tumors	4	15, 19, 21, 22	4	11, 43, 48, 15	-	
Prostate cancer	-		1	52	-	
Genitourinary	-		-		10	
**Other tumors**	8 (3.5%)^#^		13 (7%)^$^		20 (6%)^@^	

*supratentorial primitive neuroectodermal tumors, currently mainly classified as diffuse high grade gliomas, ** 4 T-cell and 5 unclassified ALL in Wimmer et al. [18] and 3 T-cell and 9 precursor B-cell ALL in Ercan et al. [10], *** multiple synchronous CRC counted as one malignancy, ^#^ other tumors: neuroblastoma, Wilms tumor, ovarian neuroectodermal tumor, infantile myofibromatosis, rhabdomyosarcoma, basal cell carcinoma, mucoepidermoid carcinoma of the parotis, osteosarcoma ^$^ other tumors: retinoblastoma, breast cancer (*n* = 2), skin cancer (*n* = 3), neuroblastoma, Wilms tumor (*n* = 2), sarcoma (*n* = 3), yolk sac tumor of ovary ^@^other tumors: retinoblastoma, breast cancer (*n* = 2), melanoma (*n* = 3), neuroblastoma, osteochondroma, pilomatrical neoplasms (*n* = 10), sarcoma, sebaceous carcinoma

The symptoms of NHL vary depending on the subtype and location and can include complaints such as coughing and shortness of breath (in the case of T-cell lymphoblastic lymphoma in the mediastinum), intestinal obstruction (B-cell lymphoma), difficulty swallowing, anemia, fatigue, and petechiae.

A recent international collaborative study involving C4CMMRD, IRRDC and European Intergroup for Childhood NHL (EICNHL), identified 74 patients with CMMRD-associated NHL of which one third had been previously reported. The median age at diagnosis was 9.4 years and the 5-year survival rate was 61.5%. A high proportion of patients (20 (27%) of the 74 patients) developed multiple NHL [28].

ALL presents with symptoms such as anemia, thrombocytopenia, neutropenia, lymphadenopathy, bone pain, fever, and bleeding. Ercan et al. reported a 10-years overall survival rate of 67% for 61 CMMRD-patients with hematological cancers [10]. Hypermutability leading to antigen-escape and thereby impacting treatment with monospecific antigen targeting therapeutics for advanced disease is a challenge that needs to be explored in future studies [29].

### Lynch syndrome-associated tumors in the second decade of life

From adolescence onward, patients with CMMRD primarily develop tumors that are also associated with Lynch syndrome, such as colorectal and small bowel carcinoma (Table 1).

Colorectal carcinoma is diagnosed at a median age of approximately 16 years. Patients develop colorectal adenomatous polyps at a median age of 9 years, with cases reported as early as age 6 [20]. Polyp burden is highly variable, ranging from a few lesions to over 100 polyps. Importantly, colorectal polyps in CMMRD may exhibit a more aggressive phenotype than those in Lynch syndrome, with more rapid progression to carcinoma [20].

Small bowel carcinomas arise with a median age at diagnosis of 21–28 years (Table 1),while small bowel adenomas develop at a median age of 12 years [20]. Nonspecific symptoms such as abdominal pain, bowel obstruction, and anemia from occult blood loss can also frequently lead to a late diagnosis in these cases. Ercan et al. identified 75 CMMRD patients with gastrointestinal cancer and reported a favorable 10-years survival rate of 89% [10].

### Other tumor types

Beyond the tumors already mentioned, other types occur in 3.5-7% of cases, particularly in young adults (Table 1). These tumors included neuroblastoma, Wilms tumor, ovarian neuroectodermal tumor, infantile myofibromatosis, rhabdomyosarcoma, osteosarcoma, sarcoma, mucoepidermoid carcinoma of the parotis, retinoblastoma, breast cancer, yolk sac tumor of ovary, osteochondroma, pilomatrical neoplasms, basal cell carcinoma, sebaceous carcinoma and melanoma. The full spectrum of these associated tumors is still being characterized through a collaborative study by IRRDC and C4CMMRD, which will assess whether surveillance guidelines need modification for adults with CMMRD.

### Non-neoplastic manifestations

Non-neoplastic manifestations are also frequently observed in CMMRD. These include café-au-lait spots, which can lead to a suspected diagnosis of neurofibromatosis type 1 (NF1) [30]. In the case report series of Wimmer et al. [18], 27 (18%) of 146 patients had café au laits spots and other signs reminiscent of NF1. Ercan [10] reported that combining with other manifestations, clinical criteria for neurofibromatosis type 1 were fulfilled in 35 (28%) of 126 patients with CMMRD who had complete skin data. Guerrini-Rousseau et al. described a patient initially clinically diagnosed with NF1 who developed a glioblastoma [31]. Genetic analysis revealed a biallelic germline *PMS2* PV and a postzygotic *NF1* PV. Moreover, among a series of 22 CMMRD patients, they identified two additional patients with *NF1* mosaicism. These data support the hypothesis that *NF1* is a target of MMR deficiency and that, in some CMMRD patients, NF1-associated phenotypic features in CMMRD patients are a consequence of early *NF1* mutations.

Other manifestations include areas of other skin hyper- and hypopigmentation, multiple developmental venous anomalies (DVA), agenesis of the corpus callosum, cavernous hemangiomas, mild immunoglobulin deficiencies and rarely pediatric systemic lupus erythematosus (SLE) [23, 32–35].

## Molecular pathogenesis: DNA-mismatch repair (MMR)

The MMR genes play a crucial role in correcting replication errors, including mismatches and insertions-deletions in microsatellites. When pathogenic variants in MMR genes disrupt this repair mechanism, it leads to an accumulation of mutations. In Lynch syndrome, these mutations particularly affect genes containing microsatellite sequences, such as *TGFβRII*, *ACVR2A*,* MSH3/6*, and *BAX*, resulting in dysregulated cell growth and tumor formation [36]. In CMMRD, MMR deficiency caused by pathogenic biallelic germline variants is present from the zygote stage in all cells. This leads not only to more frequent somatic mutations in the microsatellite-containing genes mentioned above but also to somatic point mutations in other genes, notably *POLD1*, *POLE*, *TP53*, *ATRX* and *NF1* [10].

It may be hypothesized that the unique CMMRD tumor spectrum—characterized by early-onset T-cell lymphomas and high-grade gliomas—stems from the confluence of systemic MMR deficiency and intense cell division during early development. Tissues with high proliferative activity in the first years of life, such as the thymus and the brain (with its population of gliogenic progenitor cells), are particularly vulnerable to accumulating replication-associated mutations, thereby predisposing to these specific cancers [37, 38]. This pattern contrasts sharply with Lynch syndrome (LS), where MMR deficiency is acquired through a somatic “second hit” later in life. By adulthood, the thymus has involuted and gliogenesis has slowed, explaining the rarity of these tumors in the LS population [39].

However, recent studies highlight that LS-associated high-grade gliomas may be more common in childhood than previously recognized. Friker et al. identified MMR deficiency in 10 of 127 (8%) pediatric high-grade gliomas; only one was attributed to CMMRD, while six were confirmed as LS [40]. A larger study by Ngem et al. analyzing 1,389 gliomas in children, adolescents and young adults found MMR deficiency in 3.7–12.4% of high-grade cases. Crucially, of 33 MMR-deficient tumors identified, 20 were associated with LS and 13 with CMMRD [41]. This apparent predominance of LS is likely explained by its vastly higher population prevalence, estimated to be ~ 3,000 times more common than CMMRD.

To better understand the mutagenic processes in CMMRD, recent studies have analyzed large tumor series using whole-genome sequencing and the LOGIC assay [10, 42]. All tumors exhibited mutational and microsatellite signatures of MMR deficiency, confirming it as the fundamental driver of tumorigenesis. The studies reported variable albeit high tumor mutational burdens (TMB) in all tumors, with co-occurrence of somatic mutations in *POLE* and *POLD1* leading to TMB > 100 mutations/Mb (ultra-hypermutation). The highest TMBs were observed in central nervous system (CNS) and GI cancers compared to hematological cancers. In addition, enrichments were found for deleterious *TP53*,* RAS-MAPK* pathway, and *ATRX* mutations. The authors suggested that ultra-hypermutation caused by secondary *POLE/POLD1* mutations explains the frequent *TP53* loss, as such extreme mutational burden is only viable in cells that have also acquired *TP53* loss (which abrogates cell cycle checkpoints). *ATRX* mutations may further contribute to genome instability and are enriched in the same tumor type [10, 43–45]. Furthermore, Ercan et al. found that CNS tumors had very high TMBs but relatively lower “MMRDness” (microsatellite indel accumulation). Hematological tumors had lower TMBs but higher MMRDness, while GI cancers had high values for both. These differences are probably caused by tissue-specific factors like cell division rate and the acquisition of specific secondary mutations [10].

In CMMRD, the distribution of biallelic pathogenic variants varies across series. Ercan et al. (*n* = 201) reported *PMS2* in 65%, *MSH6* in 26%, and *MLH1/MSH2* together in 8% [10]. Wimmer et al. (*n* = 146) found *PMS2* in 58%, *MSH6* in 20%, and *MLH1/MSH2* in 22% [18]. Although the prevalence of tumor types per MMR gene differs between studies, a trend indicates that tumors develop earlier in carriers of *MLH1* or *MSH2* pathogenic variants compared to those with variants in *PMS2* or *MSH6* [10, 18]. There is also evidence that carriers of *MLH1* and *MSH2* variants have a poorer prognosis [10]. This study also highlights a genotype–phenotype correlation depending on the type of variant, particularly with an earlier age at first-tumor diagnosis in the case of truncating variants, and better survival when at least one of the two variants is a missense type, at least for *PMS2*. Recently, biallelic deletions in *EPCAM*, a gene upstream of *MSH2*, have been described as a rare cause of a tissue-specific form of CMMRD. In contrast to classical CMMRD, affected individuals develop only gastrointestinal tumors without brain or hematological malignancies, reflecting *EPCAM*’s exclusive expression in epithelial cells [46].

## Indications for genetic testing of CMMRD

CMMRD should be considered particularly in children with high grade glioma, lymphoblastic lymphoma, colorectal cancer or multiple colorectal adenoma (after excluding *APC/MUTYH* polyposis), but in principle any cancer in a pediatric or young adult patient may be associated with CMMRD. The diagnosis of CMMRD is often missed due to its low prevalence and recessive inheritance. A contributing factor is that in more than half of patients, the disease is caused by biallelic *PMS2* variants. Because heterozygous pathogenic *PMS2* variants are associated with a low cancer risk, the family history for Lynch syndrome-associated tumors in these patients’ families is often negative. Furthermore, the parents (heterozygous carriers of the pathogenic MMR variant) are typically still young and healthy when their child develops clinical symptoms.

In 2014, the C4CMMRD group established diagnostic criteria for genetic testing; according to these criteria, scoring points are assigned to cancer patients based on the presence of specific clinical features [18]. If the score reached three or more points, genetic testing is indicated. In 2024, the criteria were updated as part of guideline development via the European Reference Network (ERN) GENTURIS [23]. The new criteria for testing are shown in Table 2.

In addition to clinical criteria, a high TMB in a tumor from a patient under 18 years of age, or immunohistochemical loss of MMR protein expression in both neoplastic and non-neoplastic tissue, as well as identification of a pathogenic MMR gene variant through tumor sequencing are also indications for genetic testing.

**Table 2:** Revised C4CMMR indication criteria for CMMRD testing in cancer patients. Source: ERN GENTURIS guidelines: Colas et al. [23] (www.genturis/cmmrd-guideline.htlm)

CMMRD testing is indicated in a cancer patient reaching *≥* 3 points	
	Points
**C4CMMRD scoring points assigned to (pre-) malignancies in the patient (at least one point is mandatory): **	
Carcinoma of the Lynch syndrome (LS) spectrum^a^ and/or a high-grade dysplastic adenoma of the digestive tract at age < 25 years	3
Multiple colorectal adenomas at age < 25 years and no genetic diagnosis/explanation upon testing for polyposis syndromes	3
T-cell lymphoblastic lymphoma at age < 18 years	2
WHO grade III of IV glioma at age < 25 years	2
Any other malignancy at age < 18 years	1
**C4CMMRD scoring points assigned to additional features in the patient (optional): **	
Clinical sign of Neurofibromatosis type 1 (NF1)^b^- and/or *≥* 4 hyperpigmented and/or hypopigmented skin alterations with diameter> 1 cm	2
2 or 3 hyperpigmented and/or hypopigmented skin alterations with diameter > 1 cm (do not count if two points are already given for previous criterium)	1
Multipele pilomatrixomas	2
One pilomatrixoma	1
Agenesis of the corpus callosum	1
Non-therapy-induced cavernoma	1
Multiple developmental venous anomalies (DVAs, also known as cerebral venous angiomas) in separate regions of the brain	2
Paediatric systemic lupus erythematous	1
Deficiency/reduced levels of IgG2/4 and/or IgA	1
**C4CMMRD scoring points assigned to additional features in the family (optional) **	
Consanguineous parents	1
Diagnosis of LS in a first-degree or second-degree relative	2
Carcinoma from LS-spectrum^a^ before the age of 60 years in a first-degree, second-degree, and/or third-degree relative	1
A sibling with a (pre) malignancy assigned two or three C4CMMRD scoring points	2
A sibling with any type of childhood malignancy	1

^a^ Colorectal, endometrial, small bowel, urothelial, gastric, ovarian, and biliary tract cancer

^b^ Clinical sign in the patient used for the diagnosis of NF1 according to: Legius et al. Revised diagnostic criteria for neurofibromatosis type 1 and Legius syndrome: an international consensus recommendation. Genet Med 2021;23(8):1506–1513

Furthermore, genetic testing for CMMRD can be indicated for patients who present with NF1-like features but lack a pathogenic germline variant in *NF1* or *SPRED1*, and have no family history of confirmed NF1. Considering the rarity of CMMRD in this group of patients (0.41%) [47], additional criteria that should be fulfilled for testing in this context have been established [6, 23].

The new guidelines also address CMMRD testing in Lynch syndrome families. Testing for partners of patients with Lynch syndrome is recommended if there is consanguinity, a positive family history for LS carcinomas in the partner, or if the partner originates from a population with a known founder mutation. Also, Lynch syndrome patients with a carcinoma diagnosed before the age of 18, should be tested to exclude the presence of a second PV that was not previously known in the family [23].

Finally, according to the new guidelines, in cancer patients with a suspected diagnosis of CMMRD that cannot be confirmed, testing for germline *POLE* and *POLD1* exonuclease domain variants should probably be considered as certain germline PV in *POLE* and *POLD1*, and combinations of *POLE* or *POLD1* and MMR gene PV can cause a condition similar to CMMRD [48, 49].

## The contribution of new “ancillary” tests to confirm the diagnosis of CMMRD

The C4CMMRD guidelines recommend a strategy aimed at achieving a definite diagnosis or exclusion of CMMRD. Classical fragment length analysis-based MSI testing is not recommended, as it frequently fails to show MSI in ultra-hypermutated tumors. If tissue is available, immunohistochemistry (IHC) of the MMR proteins should be performed, even though IHC analysis has limitations, including poor tissue quality, missense variants with retained expression, and interobserver variability.

Unfortunately, molecular genetic testing is not always conclusive, and the diagnosis of CMMRD is frequently confounded by MMR variants of unknown significance (VUS) and *PMS2* pseudogenes. In the last decade, multiple sensitive tests referred to as “ancillary tests” have been developed that detect microsatellite instability in non-neoplastic tissue (e.g. blood) and can therefore be helpful in such cases [22, 23].

Ingham et al. [50] were the first to demonstrate germline MSI (gMSI) detection, but their dinucleotide repeat method was ineffective for *MSH6*-related cases. Bodo et al. (2015) subsequently developed a validated functional test using lymphoblastoid cell lines, which successfully diagnosed CMMRD across all genes and is currently used in routine diagnosis [51].

Later, Shuen et al. [52] created an in vitro assay with high specificity and sensitivity, though its reliance on live cell cultures limited its scalability. Addressing this need, Gallon et al. [53, 54] presented a simple, scalable, and cost-effective assay using next-generation sequencing (NGS) to detect low-level MSI in blood, suitable for both, highly sensitive, specific and rapid diagnosis and for population screening. Similarly, Gonzalez-Acosta et al. [55] and Fatima Marin [56] used sequencing to develop a high-sensitivity gMSI test, confirming its accuracy.

All of these NGS-based assays have demonstrated > 99% sensitivity and specificity for CMMRD diagnosis.

Most recently, Chung et al. [57] reported the “MMRDness” test (or LOGIC assay). This method uses low-pass whole-genome sequencing to generate a score that reflects the total genomic microsatellite indel burden, also achieving nearly 100% sensitivity and specificity for CMMRD diagnosis.

In conclusion, the high performance of these tests allows to discriminate between CMMRD and other clinically related syndromes and may confirm the diagnosis in cases with uncertain genetics testing results including cases of ‘likely CMMRD’. The integration of these test results with clinical phenotype and genetic data has led to the development of formal diagnostic criteria. While earlier guidelines [13, 22] proposed both “definite” and “likely” diagnostic categories, the latest recommendations developed by ERN GENTURIS in collaboration with C4CMMRD [23] advise against using “likely” criteria emphasizing the need for conclusive evidence given the serious implications of a CMMRD diagnosis.

## Genetic counselling and psychosocial support

According to the ERN GENTURIS guidelines, during the diagnostic process, genetic counseling should be offered to the parents and siblings of the patient by a multidisciplinary team with expertise in CMMRD. This counseling should discuss the advantages and disadvantages of the possible outcomes of genetic testing (diagnosing Lynch syndrome or CMMRD), as well as the options for surveillance, prenatal testing, and preimplantation genetic testing. Healthcare teams must be aware of the profound psychosocial implications of a CMMRD diagnosis. Given its significant impact on the entire family, early and continuous psychosocial support is an integral part of the care pathway [23].

## Surveillance outcomes from consortia studies

CMMRD is a severe tumor predisposition syndrome in which various aggressive tumors develop at a young age. Without timely intervention, most patients do not survive their first decade of life. To improve survival rates, structured surveillance programs aimed at the early detection of tumors have been developed [19–21]. Recent studies show that this approach leads to a significant improvement in prognosis.

A first large-scale study, conducted by the IRRDC collected data from 110 CMMRD patients worldwide [11]. From this group, 53 patients participated in a surveillance program. During follow-up, a total of 61 tumors were detected, a significant proportion of which were asymptomatic: 20 brain tumors (15 of which were without symptoms), 24 digestive tract tumors (all asymptomatic), 12 hematological malignancies (only 2 asymptomatic), and 5 solid tumors (also without clinical signs). In addition, 64 benign and low grade lesions were detected, mainly gastrointestinal polyps and low-grade gliomas. The most striking result was the difference in survival: patients who followed the full surveillance program had a 4-year survival rate of 79%, compared to only 15% in non-participants.

A second prospective study, conducted by the European C4CMMRD group, followed 22 patients for an average of four years [58]. Eighteen of the 22 patients had already developed 27 malignancies prior to the start of the study. During the surveillance period, 15 new tumors were diagnosed. This included 8 asymptomatic tumors—comprising 3 brain tumors, 3 tumors in the upper digestive tract, and 2 colorectal tumors—and additionally 7 symptomatic tumors, which included 2 brain tumors, one small bowel cancer, and 4 hematological malignancies. Moreover, adenomas were detected and subsequently removed involving colorectal adenomas in 12 patients and duodenal adenomas in 6 patients. Despite the complexity of these cases, the majority of tumors were successfully treated. After four years, 73% of the patients were still alive.

Both studies suggest that surveillance programs can improve the prognosis of patients with CMMRD. Surveillance of the digestive tract, in particular, proves to be effective, with a 5-year survival rate of 100% for patients whose tumors were discovered through surveillance [11]. Hematological tumors were not detected by the surveillance program due to the lack of an effective screening tool.

**Table 3:** Updated Surveillance protocol C4MMRD [23] and AACR working group [16]

Investigation	C4CMMRD		AACR working group	
	Starting age	Frequency	Starting age	Frequency
Clin.examination	From diagnosis	6 months	–	–
MRI-brain	2–20 yrs	6 months	At diagnosis^a^	6 months
	20 yrs	Annually		
Colonoscopy	6 yrs	Annually^b^	6 yrs	Annually
Upper-GI-endoscopy	simultaneously with colonoscopy or at least from 10 yrs	Annually	6 yrs	Annually
Videocapsule endoscopy	10 yrs	Annually	6 yrs	Annually
Gynaecological examination	20 yrs	Annually^c^	12 yrs	Annually
Transvaginal US	20 yrs	Annually^c^	–	–
Transabdominal pelvic US	20 yrs	Annually	12 yr	Annually
Transabdominal US	–	–	1 yr	6 months
Whole body MRI	At least once at diagnosis or when anaesthesia is no longer required; discuss optional annual imaging annually		6 yrs	Annually
Skin examination	–	–	At diagnosis	Annually

Abbreviations: GI, gastrointestinal; US, ultrasound; MRI, Magnetic Resonance Imaging

^a^The first MRI is recommended with contrast; ^b^ Interval should be increased to once every 6 months once polyps are detected; ^c^ Discuss prophylactic surgery once family planning is complete

Based on these studies, the C4CMMRD’s surveillance guidelines have been updated as well as the recommendations from the AACR’s Childhood Cancer Predisposition Workshop [13] as shown in Table 3. There are some differences between the recommendations, in particular, the starting age of surveillance for genitourinary cancers in the AACR guidelines [13]. The age was lowered to 12 years since the previous AACR/IRRDC-guidelines because in the most recent evaluation of the IRRDC database [10], genitourinary cancers (*n* = 10 of 339 cancer) were observed as early as at 14 years of age (without specification of cancer type or age at diagnosis). In this context, it is important to emphasize that for Lynch syndrome, the appropriate surveillance protocols and their effectiveness for urological and gynecological cancers remain unknown [59, 60].

In the new AACR-recommendations, also regular skin examination is added because of the risk of malignant transformation of benign skin tumors including pilomatrixoma, and the detection of other skin cancers. In the updated IRRDC database, 14 skin tumors were reported including 3 melanoma, 10 pilomatrical neoplasms and one sebaceous carcinoma [10].

In addition, recommendations differ regarding the use of whole-body MRI and the appropriate starting age for Video Capsule Endoscopy (VCE) (see Table 3). It is important to note that VCE carries a risk of capsule retention, particularly in patients with a history of gastrointestinal surgery. In such cases, consideration of a patency capsule prior to VCE is recommended, especially in younger or higher-risk individuals. Alternative imaging modalities, such as magnetic resonance enteroclysis (MRE), may be preferred and can also serve as a complementary tool to localise larger small-bowel polyps and guide the optimal technique for resection. Additionally, double-balloon enteroscopy plays a key role in accurately localising small-bowel polyps and is often required for their removal.

Furthermore, the surveillance interval for colonoscopy or upper GI endoscopy should be shortened if adenomas are detected. The specific interval depends on factors such as polyp size, the presence of high-grade dysplasia, and the completeness of removal. Given the potential complexity of removing large adenomas, it is advisable to perform these surveillance procedures in centers with gastroenterologists experienced in Lynch syndrome screening or, in paediatric settings, familial adenomatous poyposis.

Both proposed surveillance protocols are designed to offer a comprehensive set of investigations, based on the current understanding of cancer natural history in CMMRD. As demonstrated by Durno et al. [11], adherence to the full protocol would likely yield the best survival outcomes. In clinical practice, however, full adherence can be challenging, particularly given the young age of the patients. The associated costs may also limit participation, especially in low and middle income countries (LMIC). Therefore, the recommended approach per the ERN C4CMMRD guidelines is to: (1) discuss the individual’s cancer risks with the patient and/or parents, (2) review the advantages and limitations of the various screening modalities [19], and (3) collaboratively make an informed decision to establish a feasible, personalized surveillance plan [23]. Data from IRRDC has established that even partial surveillance as was done in many LMIC countries can improve survival as compared to not doing surveillance at all [11].

Given the significant impact of surveillance with increasing anxiety before and after the screening examinations, continuous psychosocial support is advised as an integral part of the program [23].

## Treatment

### General principles

**Fig. 1:**
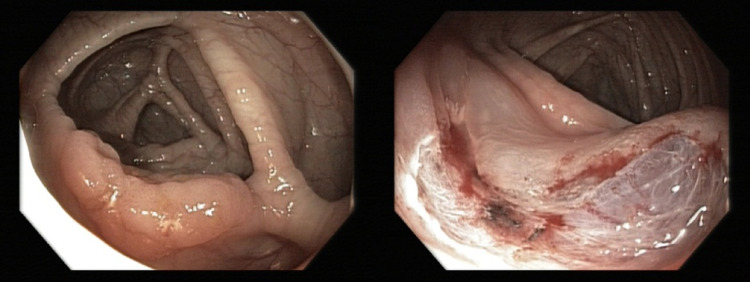
Endoscopic resection of a large sessile polyp in the colon (tubulovillous adenoma with focal high-grade dysplasia)

Treatment decisions for patients should be made within a multidisciplinary team, preferably including a specialist with expertise in CMMRD [23]. When treating patients with CMMRD, consideration must be given to the frequent occurrence of synchronous and metachronous tumors. While the prevalence of synchronous cancers is as high as 25%, biologically rational therapies can significantly improve outcomes [61]. As the median time to new cancer development is 2 years [10], ongoing surveillance and careful evaluation of current treatment choices on future management are important and often complex considerations. For example, in cases of multiple colon polyps, endoscopic resection of the polyps is often chosen over a subtotal colectomy, as the latter does not preclude risk of subsequent neoplasms in the remaining gut or other extraintestinal sites (Fig. 1).

 Upon diagnosis of a new tumor, molecular analysis of the tumor (e.g., by next-generation sequencing [NGS] of clonal markers) is indicated to distinguish between a recurrence and a new primary tumor. This is important because the treatment differs for these two possibilities. The analysis involves comparing the genetic sequences or mutation patterns between the first tumor and the new lesion. Identical mutations prove it is a recurrence, while different mutations indicate a new primary tumor.

 Due to the rarity of CMMRD, there are no randomized controlled trials available regarding the effectiveness of chemotherapy. Treatment considerations are therefore primarily based on positive experiences with specific treatments in patients with Lynch syndrome, such as the favorable response of CRC to immunotherapy [62]. Multiple studies have investigated the effects of immune checkpoint inhibitor (ICI) treatment on tumors in patients with CMMRD [15, 16, 24, 25, 27] and have demonstrated a significant impact on patient survival.

### Outcome of recent studies of immune checkpoint inhibitor (ICI) treatment

Guerrini-Rousseau et al. analyzed all brain tumors documented in the Paris C4CMMRD database to describe their clinical characteristics, management, and outcomes [24]. Among the 87 registered patients, 49 developed 56 malignant brain tumors. The study identified eight patients who received ICI treatment for a relapse of high-grade glioma, with disappointing results. Disease progression was observed within two months of initiating therapy. Six of the eight patients died a median of 5.2 months after the start of therapy, one experienced disease progression after treatment discontinuation, and one remained on therapy at the time of reporting.

 In contrast, a recent observational study from the IRRDC registry reported promising results for immunotherapy in CMMRD patients [15]. The study included 38 patients with predominantly recurrent or progressive tumors, comprising 31 CNS tumors, 11 non-CNS solid tumors (10 colorectal cancers, 1 urothelial cancer), and 3 hematological malignancies. Treatment with either nivolumab or pembrolizumab resulted in objective responses in 64% of CNS tumors, 100% of non-CNS solid tumors, and none of the hematological malignancies. Patients with non-CNS solid tumors exhibited excellent overall survival (> 80%), while those with recurrent/progressive CNS tumors also demonstrated substantial survival (39.3%; 95% CI: 36.3–42.3). The authors identified high TMB and microsatellite instability (MSI) as independent biomarkers predictive of response to ICI treatment [15].

 Moreover, Das et al. [16] reported the outcomes of nivolumab treatment in a prospective clinical trial (NCT02992964) for pediatric patients with recurrent or refractory tumors characterized by MMR deficiency and/or a TMB of ≥ 5 mutations per megabase. The cohort included seven patients with MMR-deficient tumors: five with Lynch syndrome and two with CMMRD. The tumor types comprised five glioblastomas, one astrocytoma, and one colorectal cancer (CRC).

 Among the five patients with glioblastoma, the following responses were observed: stable disease in three patients, a complete response (CR) in one, and a partial response (PR) in one. Three of these patients are still alive with survival times of 35, 26, and 25 months, respectively. The other two glioblastoma patients died after 15.8 and 16.2 months. The patient with astrocytoma experienced progressive disease and died after 1.8 months. The patient with CRC achieved stable disease by trial criteria, yet achieved a pathological complete response, and remains alive after 53 months.

 Subsequent work by IRRDC has demonstrated the efficacy of combinatorial treatment on an immunotherapy background using dual checkpoint and/or combined targeted and checkpoint inhibition, alongside re-irradiation, in patients with glioblastoma refractory to anti-PD1/PDL1 monotherapy [17]. This was based on the discovery of unique resistance mechanisms prevalent in these genomically unstable cancers.

 Recent work identifying distinct high-grade glioma subgroups [25] has paved the way for developing subgroup specific precision clinical trials based on genomic and immune biomarkers and risk-profile, including the recently initiated NCT06519682 trial that aims to spare or delay radiation in a subgroup of favorable-risk patients.

### ERN GENTURIS-C4CMMRD consortium treatment guidelines

According to the European guidelines, resection is advised in the case of low-grade gliomas [23]. For high-grade gliomas, temozolomide in CMMRD should be avoided, and immunotherapy should be used, as supported by the data presented above [23].

 CMMRD patients with NHL, and leukemia can likely be treated in the same way as patients with these tumors who do not have CMMRD. The group also states there is no contraindication for the use of radiotherapy and hematopoietic stem cell transplantation in CMMRD. However, allogenic stem cell transplant may make subsequent checkpoint-inhibition therapy for subsequent cancers more challenging. This needs to be discussed in expert groups before embarking on treatment.

 Immunotherapy is also recommended for CMMRD patients with unresectable or metastatic CRC and other LS-associated tumors. For other (non-LS) tumors, this therapy can also be considered if an alternative treatment is unlikely to be successful.

### Preventive strategies

 In Lynch syndrome, treatment with aspirin has been shown to reduce colorectal cancer incidence [63]. However, the implementation of aspirin prophylaxis in CMMRD patients remains controversial due to higher risk of brain tumor and DVAs in the brain. The ERN GENTURIS guidelines do not recommend the use of aspirin in CMMRD [23]

 Immune-based strategies, including preventive vaccines and a trial aimed at delaying or even preventing the occurrence of a second malignant tumor through anti-PD1 treatment, are being developed by the IRRDC and C4CMMRD consortia.

## Conclusion

 The concerted efforts of international consortia have substantially advanced the care of patients with CMMRD. Initial results from the implemented surveillance programs are promising. Recent developments in relatively inexpensive and accurate assays for detecting microsatellite instability (MSI) in blood cells offer significant potential. These tests can not only confirm the diagnosis in uncertain cases but could also facilitate the identification of CMMRD patients by screening all children presenting with high-grade gliomas, hematological malignancies, or gastrointestinal cancers. As these assays are relatively low-cost, they represent a viable tool for less-resourced settings, though constitutional MSI analysis should be carried out by reference laboratories. Currently, the most promising developments are emerging from the IRRDC, reporting on the efficacy of immunotherapy. This approach has shown significant benefit in CMMRD-associated colorectal cancers and other solid tumors, as well as encouraging results in a subset of patients with high-grade gliomas.

 Looking ahead, global initiatives such as the ongoing efforts of the IRRDC and C4CMMRD, aim to characterize the adult tumor spectrum and establish evidence-based surveillance guidelines for this emerging patient group. Future research will further refine care through novel applications of circulating tumor DNA (ctDNA) for monitoring and will explore next-generation strategies, including preventive vaccines and interventions, ultimately paving the way for a more proactive and personalized model of CMMRD management.

## Data Availability

No datasets were generated or analysed during the current study.
